# Promoting the social value of research in Kenya: Examining the practical aspects of collaborative partnerships using an ethical framework

**DOI:** 10.1016/j.socscimed.2008.02.016

**Published:** 2008-09

**Authors:** Geoffrey Mbaabu Lairumbi, Sassy Molyneux, Robert W. Snow, Kevin Marsh, Norbert Peshu, Mike English

**Affiliations:** a Kenya Medical Research Institute, Centre for Geographic Medicine Research Coast, Wellcome Trust Research Programme, P.O. Box 43640, Nairobi, Kenya; b Kenya Medical Research Institute, Centre for Geographic Medicine Research Coast, Wellcome Trust Research Programme, Kilifi, Kenya

**Keywords:** Kenya, Ethics, Social value, Research benefits, Collaborative research

## Abstract

The ethics of research continue to attract considerable debate, particularly when that research is sponsored by partners from the North and carried out in the South. Ethical research should contribute to social value in the country where research is being carried out, but there is significant debate around how this might be achieved and who is responsible. The literature suggests that researchers might employ two inter-related strategies to maximise social value: collaborative partnerships with policy makers and communities from the outset of research, and dissemination of research results to participants, policy makers and implementers once the research is over. These areas have received relatively little empirical attention. In this study, we carried out 40 in-depth interviews to explore the role of collaborative partnerships in health research priority setting, and the way in which research findings are disseminated to aid policy making and implementation in Kenya. Interviewees included policy makers, researchers, policy implementers and representatives of organisations funding health reforms in Kenya. Two policy issues were drawn upon as tracers wherever possible: (1) the introduction of Artemesinin- based Combination Therapies (ACTs), an anti-malarial treatment policy; and (2) *Haemophilus influenzae* (Hib) vaccine for the prevention of pneumococcal diseases among children.

The findings point to significant gaps in the ‘research to policy to practice’ pathway, particularly for national research institutions with a focus on clinical/biomedical research. These gaps reflect poorly effective partnerships among stakeholders and limit the potential social value of much research. While more investment is needed to establish strong structures for promoting and directing collaboration and partnership, how to target this investment is not entirely clear, especially in the context of the considerable power of the global health agenda and the research financing tied to it.

## Background and introduction

Discussion of the ethics of research involving human subjects often focuses on research design and approval, and on how individual research participants' rights and welfare are to be protected during health research. However, as research sponsored by government agencies, foundations, and private companies in developing countries has increased, growing attention is being paid to the ethical questions that arise when a study is concluded. These questions include: what, if anything, should be provided to research participants, and by whom, after their participation, and what, if anything, should be made available to others in the host community or country following completion of the research (Benatar and Singer, 2000, Bhutta, 2004, Emanuel et al., 2000, Nuffield Council on Bioethics, 2005, Ogundrin, 2004). These questions raise complex ethical, social and policy issues.

It is now widely recognized that an important ethical aspect of research practice ought to be the consideration of its capacity for generating social value locally through the generation of knowledge that can lead to generalized health improvements (Emanuel, Wendler, Killen, & Grandy, 2004). The importance of considering social value has gained prominence for several reasons. First, the perceived potential benefits of research to the entire population are increasingly used as a powerful justification for conducting different types of health-related research. Second, there is an increasing realization that most research does not get into practice and that translating research into health improvements is complex, incremental and haphazard (Graham et al., 2006, Kerner, 2006, Lavis, 2006, Pablos-Mendez and Shademani, 2006). Although this is not unique to developing countries, the situation is exacerbated by weak health care infrastructure and resource constraints (Black, 2001). Third, there is a concern that in the absence of an infrastructure that translates research results into health system improvements in developing countries, it is individuals or communities that assume any risks of research while the benefits accrue either to researchers themselves, through career enhancement, or to research sponsors who are recognized for their scientific profile (Benatar et al., 2005, Emanuel et al., 2004). Most international guidelines on the conduct of research are relatively silent on the issue of how the social value of research might be promoted. This poses particular challenges for researchers, ethical review committees, sponsors and regulatory bodies especially where the justification for research is in part or whole to obtain benefit for the wider population (Grady, 2004, Grinyer, 2001).

Emanuel et al.'s recent paper distils existing ethics documents to draw up a framework to assist researchers and research ethics committees assess how well ethical principles are being fulfilled in practice in developing countries. They suggest considering a series of benchmarks in relation to eight key ethical principles. Social value and collaborative partnership are the two principles that focus on the public good/benefits coming from research, as opposed to more individual considerations. Special attention is given to the principle of collaborative partnership in low income settings. It is premised on the need to minimise the possibility of exploitation of research participants by ensuring that researchers and sponsors from developed countries work together with local researchers, policy makers and communities in the developing world to determine the health priorities that should be the subject of research. In addition, this partnership is meant to provide a conduit through which research findings can influence policy decisions and practice. Ultimately, therefore, research findings should continue to strengthen the partnership by helping to define problems and priorities – part of their social value. Four benchmarks are proposed for judging the potential social value of research and five for the collaborative nature of partnerships (Table 1).

**Table 1:** Ethical principles and benchmarks for multinational research (Emanuel et al., 2004)

Principle	Benchmarks
Collaborative partnership	• Identify local partners • Shared responsibility for identify importance of health problem • Respect for community value • Minimal disparities between researchers and sponsors • Fair benefits • Determine beneficiaries
Social value	• Outline potential value of research to each beneficiary • Mechanisms to enhance social value • No supplanting extant health system
Scientific validity	• The scientific design realizes scientific objectives • Study is feasible within local healthcare & physical infrastructure
Fair Selection of study population	• Selected population should ensure scientific validity • Select population to minimize the risks of research & enhance other principles • Identify & protect vulnerable pops
Favourable risk-benefit ratio	• Assess potential risks & benefits of research • Compare net risks with potential benefits
Independent review	• Ensure reviews by bodies mandated by laws and regulations • Transparent reviews by international bodies as appropriate • Ensure independence & competence of reviews
Informed consent	• Involve community in recruitment procedures & incentives • Disclose information in linguistic & culturally acceptable formats • Implement supplementary consent procedures where appropriate • Obtain consent in culturally acceptable format • Ensure freedom to participate
Respect for participants	• Develop & implement procedures to protect confidentiality • Ensure participants know their rights • Give participants information arising from the study • Monitor & develop interventions for medical conditions arising from participation • Feedback findings

Initially, our research focused on how these two principles are considered by key stakeholders in Kenya. The work later evolved to focus on one benchmark for each principle. The benchmark on shared responsibility in agenda setting, and the mechanisms used for dissemination, was chosen for collaborative partnership and social value, respectively, to explore attempts to add value to the research findings. These two were chosen due to their broad nature and likelihood of encompassing critical issues addressed by the other benchmarks under the two principles.

In Kenya, like most other countries, there are wide gaps between health research, policy and practice (Bero et al., 1998). There has been a flurry of activities in support of collaboration and partnership between researchers, policy makers and other stakeholders as a strategy of closing these gaps but these have not been extensively researched. However, even in cases where the studies have underscored the need for such partnerships, this has not been considered as an ethical imperative. The study was therefore conducted to begin to explore, using an ethical perspective, the nature of collaborative partnerships in Kenya, focusing on research agenda setting and how the efforts to disseminate findings affect their social value. The need for this exploration developed from discussions between investigators and policy makers at the Ministry of Health about the need to develop cohesive working relations between them in tackling the disease burden of interest in the country.

### Context

**Fig. 1:**
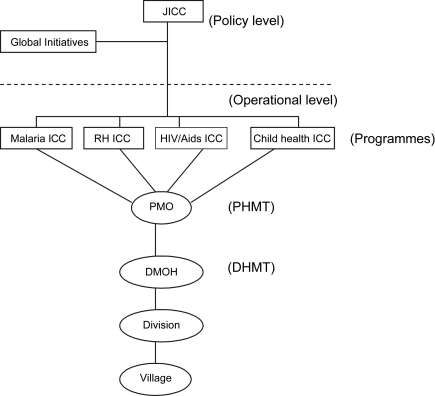
Location of inter-agency coordinating committees within the ministry of health. JICC – Joint Inter-Agency Coordinating Mechanism (a mechanism for overseeing the functions of all individual ICCs); ICC – Inter-Agency Coordinating Mechanism; PHMT – Provincial Health Management Team; DHMT – District Health Management Team; DMOH – District Medical Officer of Health; RH – Reproductive Health.

Health research is conducted in Kenya by a number of institutions comprising both government and non-governmental bodies. The government bodies include universities (under the Ministry of Education) and the institutions under the Ministry of Health (MoH), such as the Kenya Medical Research Institute (KEMRI). In theory, research by these bodies is supposed to be harmonised and integrated into the policy making process of the MoH through various inter-agency coordinating committees (ICCs) (Fig. 1), which themselves were recently established in line with key priority areas as defined in the MoH 2005–2010 strategic plan.

**Fig. 2:**
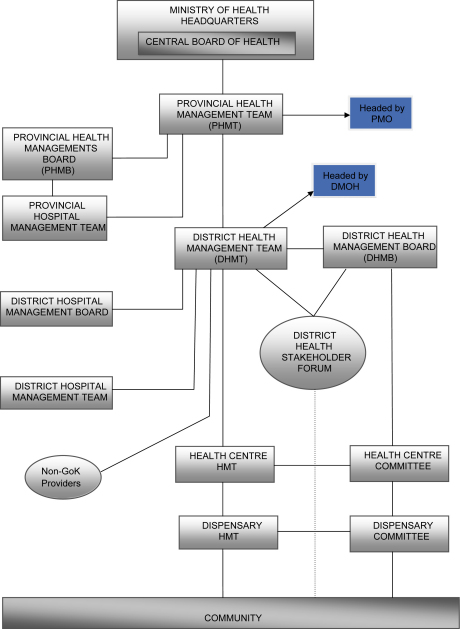
MoH levels of responsibility (adapted from Oyaya & Rifkin, 2003).

Policy implementation on the other hand is the responsibility of the provincial and district health tier (Fig. 2, Oyaya & Rifkin, 2003), under the guidance of the provincial and district medical officers, respectively. The PMOs are responsible for the interpretation, guidance and monitoring of the implementation process, while DMOHs in conjunction with the district health management teams (DHMTs) are responsible for implementation (Owino, 1997).

## Methods

### Design

The study was aimed at assembling a broad overview of the research to policy and practice interface for research findings in Kenya using an ethical perspective. While there was no intention to examine a specific policy or set of policies, where appropriate during interviews, we used two major Kenyan health policy issues as examples to help illustrate questions and ground them in practice. These findings, then, are likely to reflect the more biomedical/clinical nature of the policy tracers and organisations interviewed.

The two policy issues (tracers) were the change of anti-malarial drug policy from sulphadoxine/pyrimethamine drugs (SPs) to Artemisinin-based Combination Therapies (ACTs), and the introduction of *Haemophilus influenzae* type B (Hib) vaccine. The introduction of ACT was chosen because the decision for the policy change was made rapidly, largely on the basis of strong evidence of failure of the existing first-line drugs, but with limited locally generated efficacy data for the proposed ACT. The Hib tracer was chosen because of apparent concerns that it was pushed into the country with little collaboration and initially lacked local data to support its relevance.

The process of changing the anti-malarial treatment policy to ACTs started in 2003, following increasing resistance of the major malaria parasite (*Plasimodium falciparum*) to sulphadoxine-pyrimethamine (SP) drugs that were then the recommended first-line treatment. The recommendation by the World Health Organisation (WHO) to countries experiencing drug resistance to SP was (and is) to change to combination therapies, a view strongly endorsed by local technical and research experts. The policy change was announced by the Minister of Health in April 2004.

The Hib vaccine, on the other hand, was introduced in 2001 based primarily on evidence of vaccine efficacy from The Gambia. The initial funding commitment for the vaccine came from the Global Alliance for Vaccines and Immunisation (GAVI), with an initial agreement that the Government of Kenya would assume responsibility for vaccine financing arrangements (with partners if required) after the expiry of the initial support. This agreement assumed a widely anticipated and significant fall in the price of the Hib vaccine, an assumption that has proven to be incorrect. At the moment, the Ministry of Health has signed up for three years “bridge financing” support from GAVI, but currently there is no commitment to fund the vaccine any longer than this.

### Sampling

For this study, a broad definition of health research was used to allow inclusion of a broad spectrum of stakeholders. Health research is defined as the generation of new knowledge, using the scientific method, to identify and deal with health problems (Council on Health Research for Development (COHRED), 1990). The respondents included senior policy makers and implementers at the central, provincial and district level of the ministry of health, senior representatives from national research institutions, major non-governmental organisations (NGOs) and other bodies undertaking research, as well as bilateral bodies that help fund health reforms in Kenya. To identify participants, a list of institutions undertaking health research in Kenya was obtained from the National Council for Science and Technology (NCST), which is the oversight body responsible for authorisation and coordination of research. Non-governmental organisations were identified from the NGO coordination board, which is the body in charge of their registration. A short questionnaire was sent to these organisations, requesting information on the broad research areas that they focused on, and this was later followed with a telephone call to ensure a response. From this list, institutions were purposefully selected depending on their relevance to the topic under investigation, to include senior research scientists from KEMRI and the University of Nairobi among the national research institutions, and directors of research programmes from the Population Council, African Population and Health Research Centre, Populations Services International, Futures Group and the Family Planning Association of Kenya from among organisations involved in programme-based research. Funding agencies included UNICEF (United Nations Children's Fund), DFID (Department for International Development), and DANIDA (Danish International Development Assistance).

### Procedures and data collection

Key informant interview guides were used for data collection. These were developed after reviewing two bodies of relevant literature; one on ethics of health research in developing countries and the second on the utilisation of research in health policy making and practice. These guides were pre-tested to assess their appropriateness for collecting the intended data, and changes were made accordingly. They were, however, continually modified during the data collection to capture emerging issues. Interviews were tape-recorded and supplemented with back-up notes. Data on collaborative partnerships were elicited by asking questions on the basis for and actors involved in setting the agenda for health research, and by exploring efforts made to integrate the research findings into the health system more generally and on the two tracers in particular. Data on mechanisms used for disseminating research findings were elicited by asking participants what strategies were used for communicating or sharing their research findings with the intended users. Prior to the interviews, consent was sought from participants. The process included sending, in advance, a short description of the project explaining the study objectives, voluntary participation, right of withdrawal and the fact that comments would not be attributed to the respondents. These were further explained to them on the interview day. The principal investigator transcribed the interviews and sent back the transcripts to participants for verification. Transcripts were then stored in a password-protected database and the tapes in a secure place for back-up. In total, 40 interviews were conducted, comprising 11 policy makers, 14 policy implementers, 12 researchers and 3 health advisors.

### Data analysis

Data analysis and management were performed using NUD*IST ^N6^ (QSR International Pty Ltd., Australia). Data were explored and interrogated using a priori codes developed from our literature review. Similar data were grouped conceptually, labelled and concepts categorised and linked. An attempt was made to compare and contrast data across different categories of respondents where feasible and to further corroborate and triangulate with previous literature on ethics of research and utilisation of research in policymaking and practice. Selected transcripts were shared among three researchers who independently identified themes and later held a consensus meeting to identify the analytical themes.

## Findings

The findings are presented along two broad themes discussed in the interviews; the process and basis for agenda setting for health research, and the integration of research into policy and practice through dissemination. Specific insights from the two policy tracers are also presented, followed by a discussion on how these findings illustrate gaps in collaboration and partnership, and in promoting social value.

### Research agenda setting: who sets the agenda, on what basis and at what level?

Shared responsibility for assessing the importance of the health problem and the potential value of research to the community, planning, conducting research, disseminating findings and the integration of evidence into the health care system is one benchmark of collaborative partnership (Table 1; Emanuel et al., 2004).

Generally, there was a consensus among the different categories of respondents that the health priorities as set out in the National Health Sector Strategic Plans 1 and 2 addressed diseases with the highest burden for the country (MoH, 1999, MoH, 2005). These include malaria, HIV/AIDS/STI and TB, child health, safe motherhood and the control of environment-related communicable diseases. Our purpose, however, was to explore the links between research and health policy in more detail and the extent to which there was shared responsibility in the identification of the key issues and their prioritisation. We considered comments around research agenda setting among the four categories of respondents, namely: policy makers at the central level at the MoH, donors funding health reforms in Kenya, researchers involved in health research and actors perhaps most representative of communities, the provincial and district level policy implementers. While we did not specifically explore the role of grassroots advocacy groups, interestingly, these were not mentioned as contributing to the process of research agenda setting during our interviews.

### Health policy and research links

#### Policy makers' views

The policy makers reported that the policy agenda for health was determined based on the morbidity and mortality data from the health facilities in the country, and other sources of data such as the Kenya Demographic and Health Survey (KDHS).

…Well as far as I know, they (MoH planners) look at the statistical issues in terms of the disease burden patterns. These are derived using data from the health information systems, these random surveys and the KDHS. The provision of such data is also the responsibility of other providers in the health facilities. But whether this data is accurate or efficiently done, that's a different issue… (Policy maker 2, M, 10.03 2005).

#### Funders' views

Those funding the health reforms on the other hand said that their health policy decisions were based on evidence from specifically commissioned situation analyses and other information gathered through inter-sectoral coordinating committees.

….Our Programmes as elsewhere in the world are derived from an assessment of the country situation and also the global challenges that have to be met. For instance now there are the millennium development goals and there have also been other global challenges in the past… So based on the country situation as well as the global challenges, we agree with the government on which area of priority will be supported…(Health advisor 3, F 14. 04. 2005).

#### Policy implementers' views

Despite the reports by both policy makers and some researchers (see below) that data from the districts were used as the basis for determining the health policy agenda, policy implementers felt they were not meaningfully involved in the process:

…You see it is always made clear to us that we are not policy-makers but policy implementers and it's not our business to determine the focus of the ministry…and we have taken our position… this is the situation that we have always found ourselves in… (Policy implementer 7, M, 25.05 2005).

#### Researcher's views

Although clearly crude, it is illustrative to think of two forms of institutions conducting health research in Kenya: (1) those that are involved in both implementing health programmes and conducting research; and (2) institutions or groups whose main mandate is to conduct research in order to contribute to knowledge as a national and global public good. Programme-based health researchers reported that their health research agenda was primarily determined by their overall mandate and mission. They also mentioned, however, that efforts were made to corroborate their mandate with the local health agenda.

…It's generally the mandate of the organization… Our philosophy is that the role of our research must be practically useful to the country where we work, either at the policy or program level or just at the service delivery level…(Researcher 4, M, 31, 03 2005).

Researchers from the national research institutes reported conducting research on the priority areas already identified by the MoH:

… During the different workshops that take place in different parts of the country, we identify what are the needs and what issues are, and of course, we also use the records, although shamefully not up-to-date, the records being given by the MoH through their own annual reports, then we will be able to identify what the MoH thinks should be done….(Researcher 1, M, 12.04.2005).

… So these days, when we want to do research, we have to look at what is the direction of the MoH in terms of what kind of diseases they want to be addressed by research… (Researcher 2, M, 08.03.2005).

Nevertheless, several institute-based researchers also emphasised the importance of having a broader health research remit as opposed to only following the agenda set by the MoH:

…Research ought to do more than address priorities …we have researchers here who are experts in their own right, they understand what issues are going on in various fields, they have done research and they have published extensively in their own work and therefore they are able to understand the gaps in knowledge and then raise questions around those…So we formulate them (research priorities) through an iterative process where you address one question, come up with other questions and you look at them and extend the boundaries of knowledge (Researcher 5, M, 24.03.2005).

#### The role of global over local priorities in health policies and health research

Despite the links described above, there was a clear feeling among some policy makers and institute-based researchers that the national priorities for health, which ultimately shaped the agenda for research, were decided with heavy input from those funding health reforms in the country. Thus the national health priorities were considered to be more representative of a global rather than local health agenda:

…The need for reforms as brought out in the strategic plan was mainly pushed by the WB and WHO and the main focus was the need to redefine the health priorities for the country, seek ways and means for improving the quality of health services offered as well as ensuring the services are affordable…(policy maker 11, M, 28.06.2005).

The relative lack of power of national actors is also suggested by the frequency with which inadequacies of local health data were highlighted as a problem in setting local priorities. Data drawn from discussion about from the ACT tracer, for instance, suggest that although there were local data showing that resistance to sulphur-based anti-malarial drugs exceeded the WHO threshold, the MoH did not make the decision to adopt the ACTs until the entry of the WHO into the drug policy change arena.

… Since the year 2000, we have known that we have a mounting resistance to sulphur based drugs and it has taken us too long for the policy-makers to make a decision that we need to change. It's only when the WHO started pushing these things of ACTs that the debate started. Again you see these things are pushed more by a drug company rather than the policy-makers… (Researcher 8, M. 23.06.2005).

Some of the institute-based researchers also considered that they ought to be autonomous in the choice of research topic and to be independent in terms of the ability to challenge accepted dogma and take a longer term, more forward-thinking institutional view. It was, however, reported that this latter function was constrained, primarily by lack of resources, which led most of the research activities to revolve around the relatively resource-rich globally defined agenda.

…In my opinion, to tell you the truth, the research that goes on here is actually donor funding oriented, and of course we know that a lot of donors really look at what the country wants to do. Like currently in the areas of HIV/AIDS, malaria and TB, there is a lot of funding, and you have to write a protocol where you can get funds, and not just because it is important (Researcher 3, F, 18 .04. 2005).

### Dissemination of research findings: mechanisms and challenges

Enhancing the value of the research through dissemination of findings, product development, long-term collaboration, and/or health system improvements is one benchmark of social value (Table 1; Emanuel et al., 2004). We describe various mechanisms reported to be in place for ensuring that research findings were disseminated to policy-makers, the implementers and other category of users.

### Dissemination to policy makers

There was consensus among institute- and programme-based researchers that research findings should play a key role while addressing local health needs by directly informing policy making and supporting the implementation of such policies. Research findings were reported to be mainly disseminated through publication in journals, presentation in workshops/seminars/conferences or teaching at universities.

General mechanisms for dissemination without careful consideration and targeting of potential users at different levels was reported to be common among institute-based researchers.

… the culture is to do research and publish and forget about it, and possibly be promoted. That's where the problem really is…scientists should be educated about the need to disseminate their results in a manner that it can have impact on policy; not just for scientific journals…(Researcher 2M, 08 03 2005).

… we do not lobby the ministry of health for the adoption of our findings. We do the research and say hey look we have data here that shows this or that, what do you say about this? And then the MoH will pick from there. And even then, this information is not only provided to the MoH but also published in peer reviewed journals and it's therefore available to everybody who wants it, and they can use it to reach their own conclusions (Researcher 11, M, 19.04.2005).

Programme-based researchers were more likely to use interactive methods of dissemination, targeting specific classes of users. This is linked to their major concern of ensuring that their research output can be integrated into the existing health system. Some form of networking with policy-makers was often reported, often long before the study is complete:

…What we tend to do with ministries or academic bodies or key NGOs is to engage them more at individual levels, to try and pick key people that we should be talking to, may be at the PS level, the DMS at the MoH level or departmental heads, and just try to work with them to make sure that they understand results of research and they can use that to inform their decisions. So we do try to engage them directly, and if possible, these are the first people that we talk … (Researcher 4, M, 31, 03 2005).

It was reported, though, that these interactive methods of dissemination have many challenges. For instance, local stakeholders including the policy-makers and implementers were rarely involved while designing the study and therefore there was no guarantee that the resultant research findings addressed issues of relevance to them. In addition, these mechanisms were based on acquaintances, thereby locking out actors who are as yet not well known in the field. Third, the absence of formal structures to govern such relationships made them cumbersome and unreliable. In particular, difficulties were reported in trying to nurture and maintain informal networks beyond the life of a project or in the wake of staff turn over, a common feature of the public service:

“…You need to realize that there have been several changes within the ministry of health that we have. Three years ago, we had a different government in place and now we have different people in majority of the departments of the MoH. So maintaining those links has not been easy. Again you look at the relative cost of investing in these relationships. Even if five people in the ministry know what you are doing, what is it compared to maybe hundreds of different offices that make decisions independently, …(Researcher 5, M, 25.03. 2005).

Among the more formal mechanisms for dissemination used by researchers were presentation of brief reports to policy-makers or use of dissemination seminars with a specific invitation list including fellow researchers, policy-makers and sponsors, and the establishment of project steering committees involving policy-makers. Steering committees were used as sounding boards, and met occasionally to deliberate on emerging issues from the research. The effectiveness of these mechanisms was constrained by lack of resources to specifically address the post-research phase, the limited number of senior policy-makers that every project could target, and the inability of researchers to use jargon-free language to disseminate to potential users. On the demand side, it was reported that there was little appetite for academic research whose focus is to extend the frontiers of knowledge but often lacks immediate practical application to problem solving.

### Dissemination to participants and policy implementers

Concerns were expressed by policy implementers that researchers did not share findings with participants or even, where relevant, the facilities where research was conducted. The only exception was when policy implementers were co-opted into research projects. As a result, some of the PMOs and DMOHs reported contemplating barring the conduct of research at their facilities unless there was a commitment to disseminate findings upon completion.

…In this country, we have not only research institutes but also individuals who do research from various institutions of higher learning. Unfortunately I will say that there has not been a uniform way of harnessing research findings because these individuals when they come here, they have letter of authority from the office of the president so that we can allow them to conduct research. Most of the times, they leave after the data collection but they never ever feed back on their findings and so we don't have a data bank of research that has been conducted at the institution… I think we need to stop such kind of research from taking place here… (Policy implementer 4, M, 13 07 2005).

Policy-makers too, felt that research evidence should play an important role in supporting policy implementation. However, such sentiments were not matched by practice: no specific initiative was reported to be in place for delivering research evidence to policy implementers to support a policy action. This is despite the existence of a defined structure from the JICC to the DHMT (Fig. 1) within the MoH, intended to serve this purpose. In the case of anti-malarial drug policy change, the PMOs and DMOHs reported that the evidence behind the decision to adopt ACTs was not disseminated to them. Instead, only sensitisation seminars were reported to have been held, and used for communicating the policy direction as opposed to sharing evidence.

…I think there is very little collaboration in that field because research is done up there, they can disseminate or share the findings with the stakeholders and in this case it's generally the Ministry of Health, and they do that at the ministry level or the people from the programmes, and therefore they will form policy from there and we will be called to be updated. That's in terms of communicating policy but that's just about it and in terms of giving us research findings there is nothing… (Policy Implementer 4, M, 26.05.2005).

### Insights from the ACT and Hib vaccine tracers

Data from the two tracers provide some specific insights into the consequences of failure to negotiate adequately, the prioritisation of health problems and the impact of weaknesses in mechanisms for sharing evidence among stakeholders. In the ACT case, research evidence addressing a local problem delivered through the malaria Technical Working Group (TWG) played a key role in influencing the technical content of the policy shift from SP drugs to ACTs. In spite of this, the delays in actual policy implementation (see Box 1) illustrate the limited power of scientific evidence alone to influence decision-making, and a relative failure in recruitment of wide support for the policy shift as a result of weak mechanisms for sharing information, notably with policy implementers.

Box 1 **Anti-malaria drug policy change to ACTs**

…Well, we kept showing it [resistance to chloroquine] in our annual presentation but they didn't care until early 90s because they had not seen the clinical numbers. This is because there continued to be a wide gap between policy makers and scientists. So the scientists kept on talking about the real problems that the country was going to face and the policy makers were on the other hand saying there is no problem, we haven't seen it (Researcher 2,M, 08.03 2005).

…Since the year 2000, we have known that we have a mounting resistance to sulphur-based drugs and it has taken us too long for the policy-makers to make a decision that we need to change. It's only when the WHO started pushing these things of ACTs that the debate started. Again you see these things are pushed more by a drug company rather than the policy makers… (Researcher 8, M. 23.06.2005).

…We have technical working groups and one of them is the drug policy technical working group, and following advice from WHO asking countries that had failing drugs to re-look at the drugs with a view of adopting more effective medication, this technical group which was hitherto not been very active was revitalized (Policy-maker 6, M, 05 04 2005).

…But the adoption of the guideline (policy on ACTs) were delayed because they were pegged on the signing of the Round Four global forum on HIV/Aids, Tuberculosis and Malaria (GFATM), because this is an expensive policy, and a top policy-maker said that unless the Global Fund Round Four is signed they were not going to announce anywhere that they have accepted the guidelines (Policy-maker 6, M, 05 04 2005).

… The problem is that you read these things in newspapers even as a provincial physician like myself, nobody bothers to communicate this to us, for instance from the DOMC(Division of Malaria Control), and tell you, now this is the new malaria drug policy and that this has been supported by this and that research (Policy Implementer 3, M, 26.05.2005).

The introduction of the Hib vaccine on the other hand (Box 2) provides a sharp contrast in that initial collaboration between local stakeholders and the partners who are willing to finance the vaccine was deficient. In this case, the Global Alliance for Vaccines and Immunisation (GAVI), UNICEF and the World Health Organisation (WHO) reportedly spearheaded the introduction of the vaccine in Kenya in 2001, based primarily on evidence of vaccine efficacy from The Gambia. However, it appears that the absence of local data on or appreciation for the burden of Hib disease prior to vaccine introduction and the perceived urgency with which the vaccine was introduced all contributed to the feeling that donors pushed the vaccine into the country. Thus, although there is now emerging evidence (Cowgill et al., 2006) that the vaccine is a valuable tool to help reduce childhood mortality and morbidity, this research data may not reach an audience both receptive and large enough in time to influence a decision on its future as a component of the government immunisation programme. In a nutshell, the cases above show how lack of collaboration in the identification of key issues and lack of proper mechanisms for disseminating research findings to potential users can be a deterrent to achieving the social value of research by failing to influence policy and practice.

Box 2 **Introduction of Haemophilus influenzae type b vaccine**

…The introduction was spearheaded by GAVI, WHO and UNICEF. Their arguments were premised on the prevalence of pneumonias and meningitis disease among children and the absence of effective vaccines to counter them. This was supported by research data from West Africa, although there were no local data from this side. Of course the Ministry of Health was a bit hesitant to buy the ideas, but again there were no local data to challenge what they were saying, and you know how donors are pushy (Policy-maker 9, F, 14 042005).

…In a way, the policy-makers here, besides succumbing to donor pressure, saw an opportunity in the introduction of the vaccine in terms of infrastructure development and other related benefits (Policy-maker 8, F, 04. 04 2005).

…Personally I think there was no basis for the introduction of the vaccine here …we need more operations research… to establish what the impacts would be if we discontinued the vaccine… (Policy-maker 8, F, 04. 04 2005).

…You see we should actually be getting updates on where we are in terms of whether the pneumonias have gone down as a result of giving the vaccine but it's not there…(Policy implementer 9, M, 09.06.2005).

## Discussion

Collaboration and partnership among researchers, policy-makers and policy implementers can play a significant role in ensuring that research is responsive to a community's health problems by influencing and supporting appropriate health policies and their implementation (Council On Health Research for Development, 2001, Edejer, 1999, Swiss Commission for Research Partnership with Developing Countries, 2002, Volmink and Dare, 2005, Wilson, 1999). This can, however, only occur in the presence of strong structures for coordination and leadership of such partnerships, supported by an attendant capacity to define an appropriate health agenda among local stakeholders. A country's agenda for health provides a roadmap which informs both research and policy priority setting (Feranil, 2004). The process of setting the agenda can therefore be instrumental in closing the gap between research knowledge and practices. An exploration of the actors involved in setting the agenda for research, knowledge production and the mechanisms for sharing this knowledge in Kenya provides some critical lessons on what the gaps between research, policy and practice are, and what perpetuates them. Addressing these gaps is increasingly felt to be an ethical obligation for researchers and funding bodies.

How the agenda for research was determined more generally, and the actors involved, suggests that the considerable power of the global health agenda, often aimed at current crises, and the research financing tied to them, may inhibit the development of a more varied or context specific local agenda. This is likely to be exacerbated by the weakness of local health information, lack of local funds to support research and weak communication between those delivering health care (and the communities they serve), policy-makers and researchers (COHRED, 2002, Tangwa, 2004). Developing a more locally aligned agenda is, however, challenging. Local researchers and policy makers, for instance, can only meaningfully prioritise if they themselves are correctly informed. At present, the upward flow of information, from communities, health providers and policy implementers, remains weak. While rectifying this situation will require considerable investment it seems to be a pre-requisite to the development of a broadly based collaborative partnership.

However, there may also be an advantage if national research institutes retain some autonomy in the choice of research topic, as some independence and ability to challenge accepted dogma may provide for a longer term, more forward-thinking institutional perspective. The challenge here is the requirement for long-term funding support that is also critical for national research institutions to attract and retain local researchers, and use them in linking research to policy and practice.

Some of the current efforts to address this perennial lack of local resources include proposals for specific research budgets although the government has yet to commit to such a venture. Other efforts include high level delegations to funders (including the European Union, DFID, USAID, The Wellcome Trust and the Gates Foundation) by policy-makers, to push for the development of a funding envelope, as opposed to the current project-specific mode of funding within the health sector. The proposed mode of funding is expected to give local stakeholders some autonomy in the choice of research priorities and in the coordination of responses to those priorities. The success of these efforts will require stronger links within the national health research system, especially among the Ministry of Health, researchers, universities and those in charge of implementing health policies. In addition, agencies that fund health reforms must strive to enlarge the decision space to accommodate local stakeholders in determining local health agendas.

Whereas conducting research on identified priorities does not necessarily guarantee its influence on policy and practice (Global Forum for Health Research, 2004, Hanney et al., 2003), it is instructive to note that participants in this study did not specifically mention any attempt to bring together key stakeholders in health to collaboratively decide the agenda for health research so as to inform policy. This is despite the fact that it is widely acknowledged that such participation is key to facilitating ownership and use of research findings in influencing policy and practice (Emanuel et al., 2004, Gibbons, 1994, Pang et al., 2004). The importance of sustained and effective partnerships is underscored by our tracer examples. Although convincing evidence on the emergence of resistance to SP drugs was generated by researchers and effectively targeted to policy-makers through a MoH technical working group, policy-makers were reluctant to take any action until international financing was agreed. Once the policy and funding were in place, however, the implementers were hesitant as they were unaware of the evidence or reason for the policy change. Meanwhile, the introduction of the Hib vaccine importantly shows the danger of global decisions in the absence of structures to guide and coordinate information-based collaboration. Although these findings are not entirely surprising, they nevertheless point to lack of engagement between research, policy opinion leaders and policy implementers, and the absence of means through which such engagement can be framed within a national strategy. Most importantly, our findings indicate that policy implementers (district level actors) play an important role in the translation of research into practice, and therefore efforts to link research, policy and practice should engage with them, alongside the policy opinion leaders.

In addition to setting the agenda for research collaboratively, dissemination of findings is critical for such results to influence policy making and practice (Emanuel et al., 2004). Research findings, however, influence policy and practice in different ways (Davis et al., 2003, Lavis et al., 2003, Nutley, 2000), and information linkages between producers and potential users of research must address the specific needs of each audience. The considerable reliance on traditional means of dissemination, such as publication in international journals that are not accessible to local stakeholders (policy-makers and implementers) may compromise the potential social value of research. Dissemination strategies should be guided by a careful analysis of stakeholders who are likely to act on the basis of the available findings (Emanuel et al., 2004, Estabrooks, 2001, Lavis et al., 2002). Interestingly, dissemination of research findings within the two tracers was mainly targeted at the policy-makers, with an almost total disregard of other relevant stakeholders who may have been necessary for supporting policy formulation and implementation. At the same time, policy-makers did not consider disseminating evidence supporting a policy change to implementers and the communities at the grassroots level. These data suggest that frequent miscalculations in the range of targets for whom research has value may have been made, although it is also clear that the mechanisms which may have allowed broad dissemination are weak or non-existent. It would seem therefore that attempts to enhance the value of research should be targeted at multiple levels: dissemination to peers, policy makers, policy implementers and other potential users. This does not imply a complete mixing of levels and responsibilities but points to the need for a more interactive model of information sharing between all levels as opposed to the frequently cited linear paradigm of research to policy to practice.

The data from the two tracers also point to an ethical deficiency in the consideration of social value. It can be argued that a wider recruitment of stakeholders and broader dissemination of evidence during the anti-malarial drug policy change and the introduction of the Hib vaccine may have resulted in more informed decisions and practices and, maybe, enhanced the public good of international research. These experiences, however, raise an important ethical dilemma on just how far the responsibilities of each of the stakeholders involved in research translation extend. The dilemma has critical ramifications for the research enterprise, including problem identification, funding, actual production of knowledge and the nature of engagement between and among stakeholders.

## Conclusion

Debate on ethics of research in developing countries has previously addressed the question of what happens once research is over, with special attention paid to access to fair benefits (National Bioethics Advisory Commission, 2004, Shapiro and Benatar, 2004, UNESCO, 2005). In response, international research guidelines and principles have emphasised the need for prior agreements or collaboration and partnership among stakeholders working in health research to ensure that the prioritised questions have social value to the community by way of responding to their health needs (CIOMS, 2002, Nuffield Council on Bioethics, 2005, UNESCO, 2005). Data from this exploratory country-based study illustrate the need for greater investment to support the mechanisms and structures to coordinate the collaborative process. Importantly, our study points to a sense of isolation from the processes of agenda setting among local researchers and policy implementers and a disregard of policy implementers as a target for knowledge sharing.
